# EHMTI-0351. PFO closure with migraine in East Tallinn Central Hospital (Estonia )

**DOI:** 10.1186/1129-2377-15-S1-M12

**Published:** 2014-09-18

**Authors:** T Toomsoo, A Kaasik, S Margus

**Affiliations:** 1 Neurology, East Tallinn Central Hospital, Tallinn, Estonia; 2 Angiography, East Tallinn Central Hospital, Tallinn, Estonia

## Introduction

PFO closure may reduce the frequency and severity of migraine headaches in patients with significant right-to-left shunts.

## Methods

Between January 2008 and March 2013 consecutive patients (mean age, 34,8 years; 83.4% women) with migraine symptoms were given a prior PFO closure with the Cardia PFO occluder for migraine attack prevention. All patients had right-to-left shunts; the shunts were associated with migraine symptoms in 8 patients and all eight had aura symptoms. Septal aneurysm was present in 4 (i.e. 50%) migraine patients. All patients had very frequent migraine attacks, more then 7 times per month. All 8 patients underwent transoesophagael echocardiography after PFO closer with a clinical follow-up after 24 hr, then after 6, and 12 months, and then yearly.

## Results

An acute migraine attack occurred after PFO closure in 2 (25%) of 8 patients. There was a significant reduction (>50%) in the number and intensity of attacks in 6 (75%) of the 8 patients after the 3-month follow- up period but in 1 (12.5%) similar attacks occurred before closure. After the 12-month follow-up period , migraine had ceased in 5 (62.5%) patients, and 3 (37,5%) had a reduction in the migraine recurrence rate and disabling symptoms. These results were maintained in the follow-up (about 72 months). Only one patient remained migraine attacks, but not more than one attack per month about three month follow up period after PFO closure. There was an overall improvement in 82.5% of the treated patients.

## Conclusions

Percutaneous PFO closure in migraineurs may provide beneficial mid-term and long-term results, with significant reduction in the intensity and frequency of headache symptoms.

No conflict of interest.

